# Rapidly Progressive Bladder Cancer Diagnosed because of Spontaneous Bladder Rupture

**DOI:** 10.1155/2022/4586199

**Published:** 2022-05-09

**Authors:** Hiroki Hagimoto, Takeshi Sano, Soki Kashima, Takayuki Yoshino, Takayuki Goto, Atsuro Sawada, Shusuke Akamatsu, Toshinari Yamasaki, Masakazu Fujimoto, Yoichiro Kajita, Takashi Kobayashi, Osamu Ogawa

**Affiliations:** ^1^The Department of Urology, Kyoto University Hospital, Japan; ^2^The Department of Diagnostic Pathology, Kyoto University Hospital, Japan; ^3^Kajita Urological Clinic, Japan

## Abstract

**Background:**

Spontaneous bladder rupture (SBR) is very rare and can be associated with advanced bladder cancer. Because of its rarity, the optimal management of bladder cancer with SBR has not been established. Herein, we report a case of SBR due to locally advanced bladder cancer, which rapidly invaded the ileum and caused peritoneal dissemination. *Case Presentation*. An 86-year-old man presented with sudden-onset lower abdominal pain and distension. The patient was diagnosed with bladder perforation and bladder tumor on contrast-enhanced computed tomography (CECT). Transurethral resection of the bladder tumor revealed an invasive urothelial carcinoma with squamous differentiation. Although radical cystectomy with lymph node dissection was planned, preoperative CECT and magnetic resonance imaging revealed enlargement of the bilateral iliac regional lymph nodes, multiple peritoneal nodules, and invasion of the bladder tumor to the ileocecum. Therefore, cystectomy and resection of ileocecum with palliative intent and bilateral cutaneous ureterostomy were performed. However, the patient's general condition rapidly worsened after surgery, and he died 74 days after the initial diagnosis.

**Conclusions:**

We encountered a case of SBR accompanied by bladder cancer with extremely rapid progression, which suggested the importance of short-interval repeat imaging examinations. Emergency surgery should be considered when bladder cancer is suspected in patients with SBR so as not to miss the window period of a possible cure.

## 1. Introduction

Spontaneous bladder rupture (SBR) is very rare and is associated with trauma, pelvic irradiation, urinary retention, chronic bladder infection, malignancy, and diverticulitis [1]. It has a mortality rate of approximately 50%, although this has declined in recent years owing to increased awareness, better imaging modalities, and better management of complications [1, 2]. Bladder cancer associated with spontaneous SBR is commonly advanced at the time of diagnosis, and the prognosis is often less than 1 year [2]. Because of its rarity, the optimal management of bladder cancer with SBR has not been established. In addition, there have been no reports showing its rapid progression using serial imaging findings. Herein, we report a case of SBR due to locally advanced bladder cancer that rapidly invaded the ileum and caused peritoneal dissemination. We performed multiple imaging to confirm the rapid growth of the tumor in a short period of time (2-4 weeks).

## 2. Case Presentation

**Figure 1:**
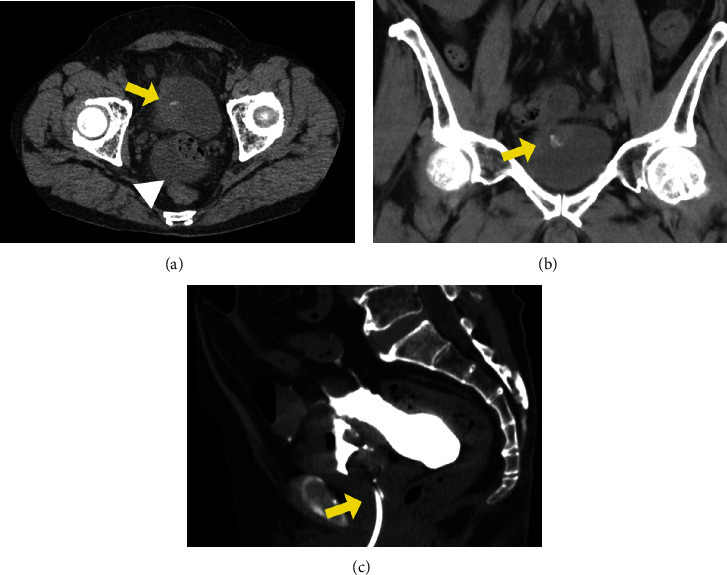
(a) An axial plain abdominal computed tomography (CT) scan showed bladder wall thickening with calcification (arrow) and ascites in the rectovesical pouch (white arrowhead). (b) A coronal plain abdominal CT scan showed bladder wall thickening adjacent to the ileum (arrow). (c) A sagittal CT scan after intravesical injection of contrast medium via a urethral catheter (arrow) showed leakage of contrast medium into the abdominal cavity.

An 86-year-old man was hospitalized in December 2019 with a chief complaint of sudden-onset severe lower abdominal pain. He had a medical history of appendicitis and no family history of notable medical conditions. Abdominal computed tomography (CT) revealed a thickening with calcification on the posterior wall of the bladder adjacent to the ileum along with ascites in the rectovesical pouch, suggesting a spontaneous bladder perforation into the peritoneal cavity potentially associated with a bladder tumor (Figures 1(a) and 1(b)). Contrast-enhanced CT (CECT) confirmed bladder perforation in the patient (Figure 1(c)). Laboratory test results, including routine blood chemistry and complete blood count, and tumor markers (AFP, CEA, CA19-9, and PSA) were not remarkable other than slight increases in SCC (2.0 ng/mL) and NSE (13.47 ng/mL). A urine test indicated slight microscopic hematuria and pyuria. Urine cytology was positive, suggesting high-grade urothelial carcinoma (UC). Microscopic hematuria was not observed during regular check-ups of benign prostatic hyperplasia before the onset of SBR.

**Figure 2:**
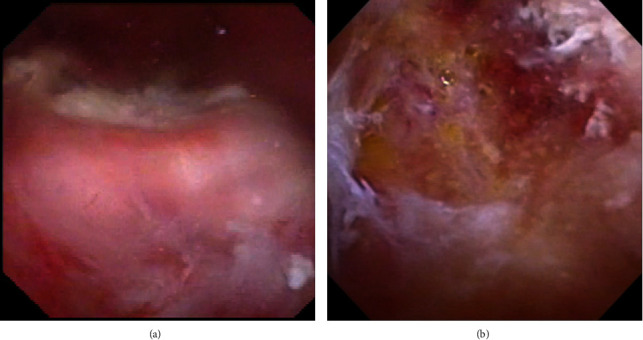
(a) Intravesical endoscopic imaging revealed an elevated nodule with whitish tissue and calcification on the surface. (b) Removal of the calcification exposed fat droplets on the bottom of the cavity.

**Figure 3:**
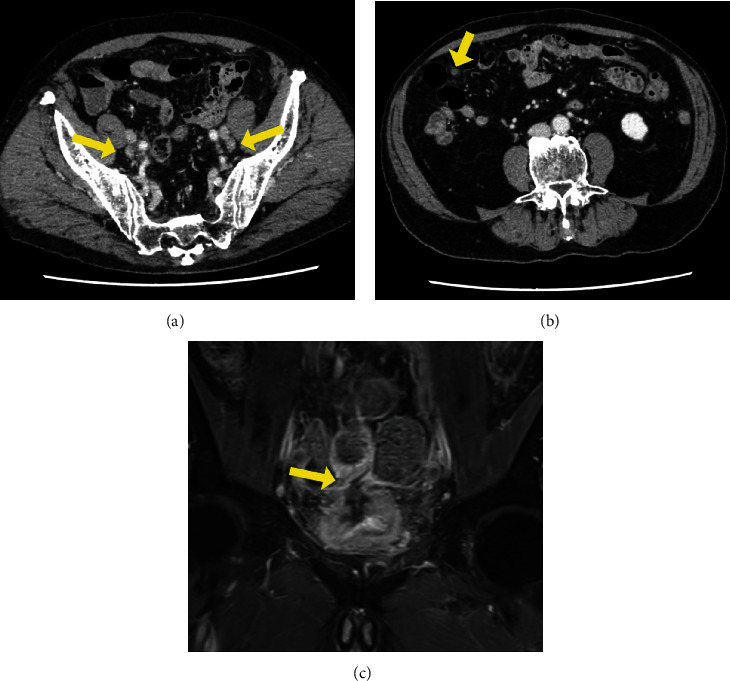
A contrast-enhanced abdominal computed tomography (CT) scan for staging showed bilateral iliac regional lymph node enlargement (a, arrow) and a small nodule in the peritoneum (b, arrow). (c) A T1-weighted TSE magnetic resonance imaging scan for preoperative local evaluation showed that the soft-tissue mass on the bladder wall extended to the small intestine (arrow), suggesting local invasion.

Transurethral resection of the bladder tumor was performed on the 2nd day of hospitalization, and an elevated lesion with a whitish surface and calcification was observed at the posterior wall of the bladder. Removal of the calcification revealed a cavity with fat droplets at the bottom of the lesion, which was likely the perforation site (Figure 2). The pathological diagnosis of the elevated lesion was high-grade UC with squamous differentiation. Intravenous administration of fluid and antibiotics was performed for the treatment of intraperitoneal infection and impaired general condition. CT on the 11th day of hospitalization with the intent of staging showed bilateral iliac regional lymph node enlargement and scattered small nodules in the peritoneum (Figures 3(a) and 3(b)). However, it was unclear whether this was peritoneal dissemination or a secondary finding from peritonitis. Magnetic resonance imaging (MRI) performed for preoperative local evaluation on the 24th day of hospitalization showed a slight enlargement of the bilateral iliac regional lymph nodes, small peritoneal nodules suspicious for peritoneal dissemination, and a new soft-tissue mass on the bladder wall extending to the small intestine suspicious for invasion (Figure 3(c)).

**Figure 4:**
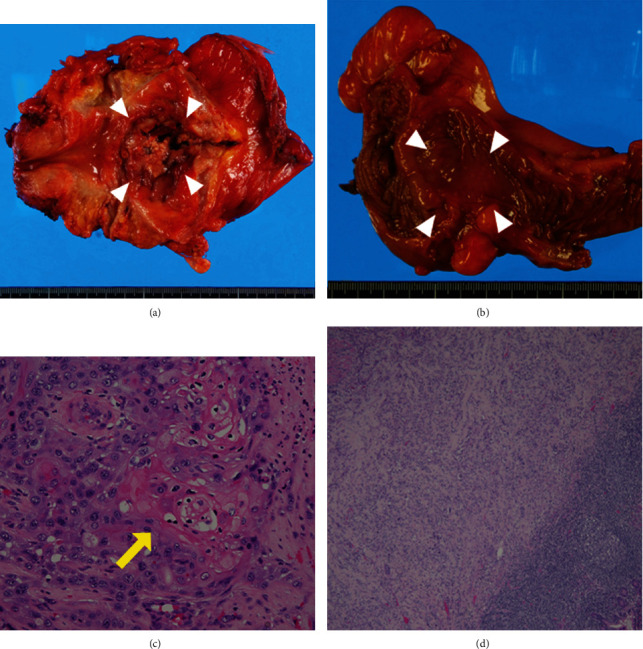
(a) A macroscopic image of the resected bladder. The bladder tumor was a raised lesion with an irregular surface, necrotic tissue, and hemorrhage (white arrowheads). (b) A macroscopic image of the lumen of the ileum separated from the bladder. There were submucosal lesions (white arrowheads), suggesting a submucosal invasion of the bladder cancer at the terminal ileum. (c) The bladder tumor was infiltrating high-grade urothelial carcinoma with partial keratinization (arrow, hematoxylin & eosin, ×200). (d) The tumor in the terminal ileum had infiltrated the submucosal layer under the normal glandular structures (hematoxylin & eosin, ×40).

Under the clinical diagnosis of cT4bN1M1b bladder cancer, open cystectomy, partial resection of the ileum, sampling of the peritoneal nodules, and bilateral cutaneous ureterostomy were performed on the 26th day of hospitalization, mainly for palliative purposes. A lower abdominal midline incision was made, and an enlarged obturator lymph node and two nodules in the greater omentum were removed for intraoperative definitive pathological diagnosis, which indicated metastatic UC. The posterior wall of the bladder strongly adhered to the ileum, suggesting infiltration of the bladder tumor, and en bloc resection of the bladder and a segment of the infiltrated ileum was performed. The specimen was a raised lesion on the posterior wall of the bladder with an irregular surface with necrotic tissue and hemorrhage (Figure 4(a)). The lesion appeared to extend to the serosa and had invaded the terminal ileum (Figure 4(b)). The pathologic diagnosis was high-grade infiltrating UC with squamous differentiation, and the pathological stage was T4bN1M1b (Figures 4(c) and 4(d)).

**Figure 5:**
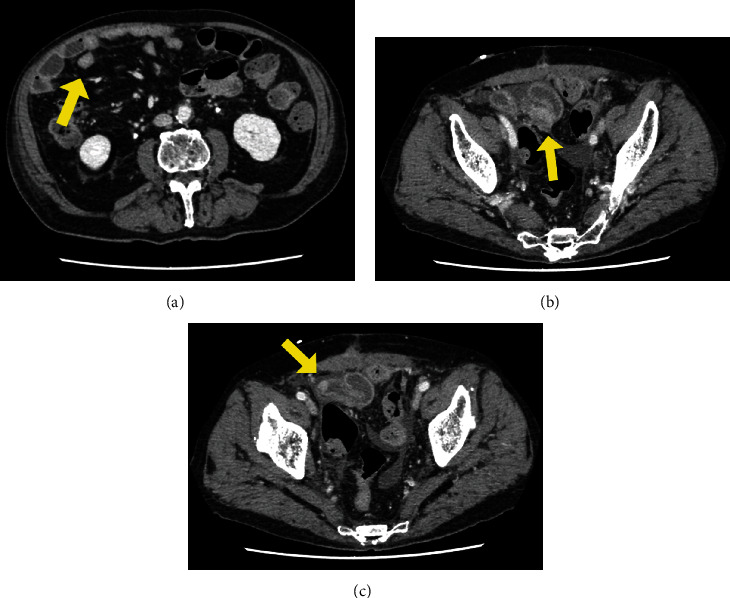
A postoperative contrast-enhanced abdominal computed tomography (CT) scan showed enlargement of one of the peritoneal nodules (a, arrow) and the invasion of nodules with edematous lumens to the small intestine (arrows) (b, c).

A CT scan on the 30th day of hospitalization showed enlargement of the left obturator lymph node and rapid growth of multiple peritoneal nodules, some of which had invaded the small intestine (Figure 5). The patient was discharged on the 58th day of hospitalization. Although systemic chemotherapy was planned, his general condition rapidly deteriorated, and he died 74 days after the initial diagnosis.

## 3. Discussion and Conclusions

SBR due to bladder cancer is very rare [2]. Because SBR itself is a life-threatening event and bladder cancer that causes bladder rupture is generally advanced, very few patients survive for more than a year, and most cases report a survival time after diagnosis from 10 days to several months [3, 4]. To the best of our knowledge, only 16 cases of bladder cancer with SBR have been reported between 2000 and 2022 including the present case (Table 1). All the cases revealed an SBR in the abdominal cavity, inferring that these patients had highly advanced tumors invading the peritoneum around the bladder. Four of these cases reported peritoneal dissemination at the time of diagnosis. Most patients died within a range of a few days to a few months, indicating a poor prognosis of bladder cancer in patients with SBR. Although approximately 90% of bladder cancers are UCs and only 2-5% are squamous cell carcinomas [5], squamous cell carcinomas cause half of bladder rupture cases associated with bladder tumors [3, 4]. In the present case, microscopic hematuria was not observed during regular check-ups of benign prostatic hyperplasia before SBR, but mild microscopic hematuria was observed at the onset of SBR, potentially suggesting the invasive nature of the tumor to the stroma without exophytic growth and the difficulty in detecting the tumor with simple clinical testing.

Although surgical repair is generally the first choice for intraperitoneal bladder rupture [6], cystectomy is required if the cause of SBR is bladder cancer. In the present case, a CT scan at the initial diagnosis suggested the existence of bladder cancer. Hence, we performed a transurethral biopsy of the elevated lesion, which revealed high-grade UC with squamous differentiation. The tumor was localized in the bladder at the initial diagnosis. However, invasion to the ileum, lymphadenopathy, and peritoneal masses were observed on CT and MRI before cystectomy. Although bladder perforation during transurethral resection of bladder tumors is associated with an increased risk of intra-abdominal dissemination, it is very rare [7, 8]. In the present case, peritoneal masses suspicious for dissemination developed only a few weeks after the onset of bladder rupture, which suggests the extremely malignant potential of the tumor. The present case is very important because serial imaging examinations in a short period, including CT and MRI, detected the “moment” of the invasion of the bladder tumor to the ileum and revealed the speed of the development and growth of peritoneal masses in cancer-related SBR, which have not been shown previously.

Owing to the recent advancements in imaging modalities, cases in which bladder cancer is diagnosed or suspected based on imaging results before surgical repair of SBR are expected to become more common. However, there is no consensus on the next step after diagnosis. In the present case, we first performed transurethral tissue biopsy followed by cystectomy based on the pathological diagnosis. Although cystectomy was performed 23 days after the biopsy, multiple metastatic tumors were found during surgery. Based on our results, we recommend emergency laparotomy for the surgical repair of SBR. However, it may not be beneficial when the general condition of the patient is already very deteriorated. Additionally, the surgery will be only palliative and may not benefit the survival of the patient. A surgical repair should be accompanied by intraoperative pathologic examination of the bladder lesions. In case the lesion is found to be nonmalignant, a simple closure of the bladder perforation should be performed. Contrastingly, if bladder cancer is found, then the patient should undergo immediate palliative cystectomy and extensive peritoneal lavage. Considering the poor survival outcome of bladder cancer with SBR, adjuvant chemotherapy may also be considered for the survival benefit, provided the patient has a good general condition postsurgery.

In conclusion, we encountered a case of SBR accompanied by bladder cancer that showed extremely rapid progression, which suggested the importance of short-interval repeat imaging examinations, although it was localized to the bladder at the initial diagnosis. Emergency surgery should be considered when bladder cancer is suspected in a patient with SBR to avoid missing the window period of a potential cure.

## Figures and Tables

**Table 1:** Summary of reported cases of bladder cancer with spontaneous bladder rupture.

No.	Year	Author	PMID	Sex	Age	Site of rupture	Peritoneal dissemination	Treatment	Histology	Outcome
1	2001	Goel A	12230278	Male	55	Dome	None	Partial cystectomy	SCC	Alive (at least 6 months)
2	2002	Jayathillake A	12561801	Female	72	Right wall	N/A	Primary closure	SCC	Dead (10 days)
3	2002	Fujiwara A	12491613	Female	65	Dome	Yes	Radical cystectomy	Sarcomatoid carcinoma	Dead (46 days)
4	2006	Shiraishi Y	16541769	Female	71	Posterior wall	Yes	Primary closure	SCC	Dead (2 months)
5	2008	Sawazaki H	19175001	Female	90	Dome	N/A	Primary closure	SCC	Dead (2 months)
6	2009	Ahmed J	19829892	Female	47	N/A	Yes	Radical cystectomy	UC	N/A
7	2010	Lee JH	20428434	Male	75	Left wall	N/A	Palliative care	N/A	Dead (3 months)
8	2012	Stojadinović M	22511430	Male	79	Dome and posterior wall	N/A	Primary closure	Micropapillary carcinoma	Dead (12 months)
9	2013	Sallami S	24227517	Male	54	Left wall	N/A	Radical cystectomy	Sarcomatoid carcinoma	N/A
10	2015	Kivlin D	26195965	Male	72	Posterior wall	N/A	Primary closure	N/A	N/A
11	2015	Kivlin D	26195965	Male	65	Dome	N/A	Primary closure	N/A	Dead (17 days)
12	2016	Oray D	27355091	Male	56	Left wall	N/A	Palliative care	N/A	Dead (1 day)
13	2018	Al Edwan GM	29852423	Male	56	Dome	None	Radical cystectomy	SCC	Dead (7 months)
14	2020	Asano T	33102109	Female	52	Dome	N/A	Radical cystectomy	UC with squamous cell diff.	Dead (10 weeks)
15	2020	Sahnoun W	33294156	Male	63	Posterior wall	N/A	Radical cystectomy	SCC	Dead (62 days)
16	2022	Present case		Male	86	Posterior wall	Yes	Radical cystectomy	UC with squamous cell diff.	Dead (74 days)

PMID: PubMed Unique Identifier; N/A: not applicable; SCC: squamous cell carcinoma; UC: urothelial carcinoma.

## Data Availability

The datasets used and analyzed during the current study are available from the corresponding author on reasonable request.

## Abbreviations

SBR: Spontaneous bladder rupture
CECT: Contrast-enhanced computed tomography
CT: Computed tomography
MRI: Magnetic resonance imaging
UC: Urothelial carcinoma.
